# Automatic radar-based 2-D localization exploiting vital signs signatures

**DOI:** 10.1038/s41598-022-11671-1

**Published:** 2022-05-10

**Authors:** Marco Mercuri, Pietro Russo, Miguel Glassee, Ivan Dario Castro, Eddy De Greef, Maxim Rykunov, Marc Bauduin, André Bourdoux, Ilja Ocket, Felice Crupi, Tom Torfs

**Affiliations:** 1 DIMES, University of Calabria, 87036 Rende, CS Italy; 2 IMEC-Netherlands, 5656 AE Eindhoven, The Netherlands; 3 IMEC, 3001 Leuven, Belgium

**Keywords:** Health care, Biomedical engineering, Computer science

## Abstract

In light of the continuously and rapidly growing senior and geriatric population, the research of new technologies enabling long-term remote patient monitoring plays an important role. For this purpose, we propose a single-input-multiple-output (SIMO) frequency-modulated continuous wave (FMCW) radar system and a signal processing technique to automatically detect the number and the 2-D position (azimuth and range information) of stationary people (seated/lying down). This is achieved by extracting the vital signs signatures of each single individual, separating the Doppler shifts caused by the cardiopulmonary activities from the unwanted reflected signals from static reflectors and multipaths. We then determine the number of human subjects present in the monitored environment by counting the number of extracted vital signs signatures while the 2-D localization is performed by measuring the distance from the radar where the vital signs information is sensed (i.e., locating the thoracic region). We reported maximum mean absolute errors (MAEs) of 0.1 m and 2.29$^{\circ }$ and maximum root-mean-square errors (RMSEs) of 0.12 m and 3.04$^{\circ }$ in measuring respectively the ranges and azimuth angles. The experimental validation demonstrated the ability of the proposed approach in monitoring paired human subjects in a typical office environment.

## Introduction

The continuous and rapid growth of the combined senior and geriatric population has resulted in an increase of age-related chronic diseases, such as congestive heart failure, chronic obstructive pulmonary disease, sleep disorders, arthritis, osteoporosis, and dementia^1^. There are currently more than 1 billion people over the age of 60 worldwide, a number that is expected to double within the next 30 years^2^. This scenario translates into a shortage of healthcare personnel, in tandem with the ever-increasing demands for healthcare services. Coupled with the expected rise in healthcare cost and given that only a minority can afford private home-care personnel, the need for technologies enabling remote patient monitoring is certainly on the rise^3, 4^.

In the last years, radar has become one of the most promising telemedicine technologies for both home and clinical environments enabling long-term smart monitoring of patients^5–11^. Practical applications are: sleep monitoring; contactless monitoring of patients in multi-bed; monitoring elderly people in domestic environment or in nursing homes; monitoring household members in quarantine or patients in departments of infectious diseases to reduce contamination risks; detecting whether people are respecting social distancing. Research focuses mainly on vital signs monitoring and indoor localization. Novel and sophisticated radars have been proposed to properly demodulate the phase shift caused by the subject’s movements (i.e., cardiopulmonary activity, walking, running, etc) and embedded into the reflected radar signal (i.e., Doppler effect). The first devices were based on continuous wave (CW) architectures^12–17^. However, they were only able to monitor one single subject without providing any information on their position. To solve this limitation, several studies have been conducted on ultra-wideband (UWB) architectures, in particular on frequency-modulated CW (FMCW), stepped-frequency CW (SFCW), phase-modulated CW (PMCW), and UWB impulse-ratio (UWB-IR) radars^18–27^. Single-input-multiple-output (SIMO), multiple-input-multiple-output (MIMO) and beamforming UWB architectures have been preferred over classic single-input-single-output (SISO) solutions as they can provide azimuth and range information, namely two-dimensional (2-D) localization, of multiple targets^28–30^. At the same time, many novel signal processing methods have been proposed to tackle the challenges that practical circumstances impose. Most research focused on monitoring a single subject. However, to better cope with many complex everyday life applications (e.g., monitoring people lying on their beds in the hospital, elderly people in nursing homes, household members in quarantine, etc), multi-people monitoring has become an important research direction. One of the biggest challenges in radar-based remote patient monitoring is to automatically estimate the number and the 2-D positions of stationary people (i.e., seated/lying down and normally breathing). With this respect, in recent years, some significant works have been published in literature. The authors proposed a SISO FMCW radar, integrating two frequency scanning antennas, which allows determining the 2-D positions and the vital signs of people^31^. Wang et al. introduced the ViMo radar sensor for multi-person monitoring with a prior knowledge of the number of subjects^32^. Su et al. presented the combination of a self-injection-locked (SIL) radar with FMCW and switched phased-array (SPA) techniques to locate multiple people^33^. Xiong et al. proposed a SIMO CW radar and an adaptive digital beamforming technique to detect the respiration and angular information of multiple individuals^34^. The information known a priori was the number of targets and their absolute distances (ranges) from the radar. Feng et al. presented a MIMO CW radar for 2-D chest motion imaging from which the angular information of each subject can be determined^35^. The system can estimate the azimuth and elevation angles where the subjects’ chests are situated but it requires the information on the number of targets and their ranges. Zhang et al. presented a SIMO SFCW radar for multi-people localization^36^. Koda et al. developed a respiratory-space clustering technique for monitoring multiple people using MIMO FMCW radar when the number and locations of people are unknown^37^. Wang et al. used a commercial MIMO FMCW radar to identify the number of multiple users and their locations^38^. Although these works achieved high accuracy in monitoring multiple individuals, one limitation is that the experiments were conducted in conditions that significantly reduce the effects of the multipath propagation^39–41^. This was achieved with no objects in between the radar and the person or with objects placed strategically to not generate significant interferences. This reduces the probability that the signals reflected from different individuals interfere each other and hence the subjects can be treated independently. If this (rare) condition is not met, especially in everyday indoor environments, those approaches would result in non-linear combinations of the Doppler signals generated by the subjects, making correlation-based algorithms unable to eliminate the multipaths^36^. This because, current approaches aim at directly extracting the Doppler signals from the received radar signals which are the overall results of the sums of reflections due to direct paths (desired information), multipath, and static reflectors (i.e., clutter, furniture, objects, etc), whose Doppler (phase) information combines non-linearly. In this condition, the radar may: (1) fail to determine the right number of subjects in the room; (2) erroneously conclude people being located elsewhere than the actual location; (3) detect a non-existing person (i.e., a radar ghost) or even represent a life-less object as alive (hence, static objects cannot be simply treated as stationary); (4) assign disturbed signals to an individual^42^.

In this article, we propose a signal processing algorithm to automatically detect the number and the 2-D position (azimuth and range) of seated people. This method, demonstrated experimentally using a millimeter-wave (mmWave) SIMO FMCW radar, is capable to isolate the Doppler signals caused by the cardiopulmonary activity (vital signs signatures) of each single subject from the reflections of static reflectors and multipaths. We also propose a phase demodulation technique based on geometric fitting which is performed of each isolated Doppler signal. The localization is then performed by measuring the linear distances from the radar where the vital signs information is sensed (i.e., locating the thoracic regions).

## Signal model and data cube generation

**Figure 1:**
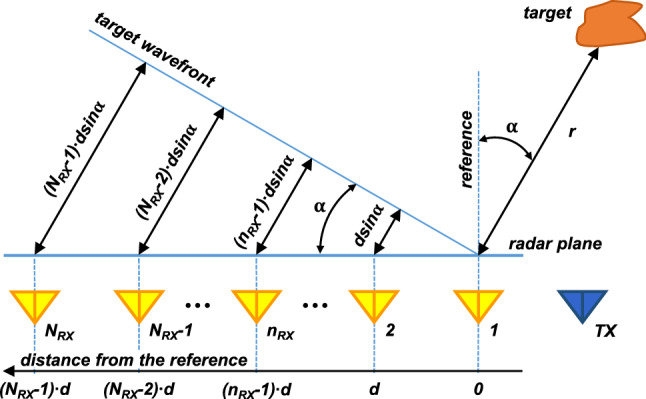
Uniform linear antenna array of a SIMO radar.

Figure 1 shows a graphical illustration of the antenna array of a generic SIMO radar with uniform equispaced linear array (ULA). The radar consists of one transmitter (TX) antenna and $N_{RX}$ receiver (RX) antennas, forming an antenna array of $N_{RX}$ elements equally spaced of *d* meters. Assuming collocated antennas and a target in the far field at a distance *r* and angle $\alpha$ from the first element of the array (considered as the reference), the signal reflected from the target should travel an additional distance of $(n_{RX}$-1)$\cdot d \sin \alpha$ to reach the $n_{RX}$-th antenna. This corresponds to a phase difference of *2*$\pi$/$\lambda \cdot$*d*$\sin \alpha$ among the signals received by adjacent antennas. Therefore, there is a linear progression in the phase of the signals across the array.

A SIMO FMCW radar radiates a series of signals, called *chirps*, whose instantaneous frequency increases linearly over time. Mathematically, a *chirp* can be expressed as:

1 $$\begin{aligned} s_T(t)=a_Te^{j2\pi \int _{0}^{t}\left( f_0+\rho t\right) dt}=a_Te^{j2\pi \left( f_0+\dfrac{\rho }{2}t\right) t }, \end{aligned}$$

where $a_{T}$ is a complex number indicating the amplitude and the initial phase, $\rho = \textit{B}/\textit{T}$ is the *sweeping rate* with *B* and *T* being respectively the total bandwidth and duration, and $f_{0}$ is the initial frequency. The *chirps* are transmitted with a certain pulse repetition interval (PRI) and are received over $N_{RX}$ multipath channels. The corresponding received signals $s_{R}(t,n_{RX},m)$ can be modelled as the convolution between the transmitted signal $s_{T}(t,m)$ and the channel impulse responses $h(t,n_{RX},m)$, as:

2 $$\begin{aligned} s_R(t,n_{RX},m) = s_T(t,m) *h(t,n_{RX},m) = \sum _{i=0} \sum _{l=0} \beta _{i,l,n_{RX}} \cdot s_T \left( t - \tau _{i,l,n_{RX}}(m)\right) , \end{aligned}$$

with

3 $$\begin{aligned} h(t,n_{RX},m)= & {} \sum _{i=0} \sum _{l=0}^{L-1} \beta _{l,n_{RX}} \cdot \delta \left( t - \tau _{i,l,n_{RX}}(m)\right) , \end{aligned}$$

4 $$\begin{aligned} \tau _{i,l,n_{RX}}(m)= & {} \left\{ \begin{array}{l} 2\dfrac{r_{i,l}+y_i(m)}{c_0}+\dfrac{(n_{RX}-1)\cdot d\sin \alpha _i}{c_0} \textit{, for subject}\\ \\ 2\dfrac{r_{i,l}}{c_0}+\dfrac{(n_{RX}-1)\cdot d\sin \alpha _i}{c_0} \textit{, for clutter/object} \end{array},\right. \end{aligned}$$

where *m* = 0, ..., *M*-1 is the *slow time* index, *M* is the number of *chirps* per TX-RX combination that should be received before starting any data processing, $\beta$ is the complex path gain which indicates the overall attenuation and phase shift, $\tau$ is the propagation path, *i* is the index corresponding to the *i*-th target/object, *l* is the path index, $\delta (\cdot )$ is the Dirac delta function, $c_{0}$ is the speed of light, and *y(m)* is the chest surface vibration caused by the cardiopulmonary activity. Equation (3) models a multipath channels as proposed by Jakes^43^ and includes the essential propagation parameters (i.e., namely magnitude, frequency and phase). Since a room has a limited size, we considered only the first *L* range bins. As consequence, we assume that the number of possible path delays is also equal to *L*. This assumption is valid since, due to the small size of a range bin, the differences in delays among the electromagnetic waves falling into a range bin are very small and translate into phase shifts. The digitized time domain *beat signals* $s_{B}(n,n_{RX},m)$, obtained mixing the received signals with the replicas of the transmitted signal of each TX-RX pair and following a low-pass filter and an analog-to-digital (ADC), can be expressed as:

5 $$\begin{aligned} s_{B}(n,n_{RX},m)= & {} \sum _{i=0} \sum _{l=0} \beta _{i,l,n_{RX}}^{*} a_T^{2} e^{j2\pi f_0 \tau _{i,l,n_{RX}}(m)} \cdot e^{j2\pi \rho \tau _{i,l,n_{RX}}(m)nT_f} \cdot e^{-\pi \rho \tau _{i,l,n_{RX}}^{2}(m)} \approx \nonumber \\&\approx \sum _{i=0} \sum _{l=0} \beta _{i,l,n_{RX}}^{*} a_T^{2} e^{j2\pi f_0 \tau _{i,l,n_{RX}}(m)} \cdot e^{j2\pi \rho \tau _{i,l,n_{RX}}(m)nT_f}, \end{aligned}$$

where $\textit{n} = 0$, ..., *N* − 1 is the index in *fast time*, with *N* being the number of samples acquired per *beat signal* and depends both on *T* and on the sampling time $T_{f}$ of the ADC. In Eq. (5), the gain (or loss) of the mixer was included in $\beta$. Moreover, the contribution of $-\pi \rho \tau _{i,l,n_{RX}}^{2}(m)$ is negligible for short-range applications as $\tau$ is in the order of few nanoseconds. After performing the Fast Fourier Transform (FFT) in *fast time*, the range profile is obtained. The resulting frequency domain signal ${X(k,n_{RX},m)}$ becomes:

6 $$\begin{aligned} X(k,n_{RX},m)= & {} {\mathscr {F}}\left\{ s_{B}(n,n_{RX},m) \cdot w(n)\right\} = \sum _{i=0} \sum _{l=0} \beta _{i,l,n_{RX}}^{*} a_T^{2} W \left( \dfrac{2\pi k}{K}-2\pi \rho \tau _{i,l,n_{RX}}(m)\right) \cdot e^{j2\pi f_0 \tau _{i,l,n_{RX}}(m)} \approx \nonumber \\&\approx \sum _{i=0} \sum _{l=0} \beta _{i,l,n_{RX}}^{*} a_T^{2} W (k)\cdot e^{j2\pi f_0 \tau _{i,l,n_{RX}}(m)}, \end{aligned}$$

with

7 $$\begin{aligned} W (k) = W \left( \dfrac{2\pi k}{K}-2\pi \rho \tau _{i,l,n_{RX}}(m)\right) , \end{aligned}$$

where *k* = 0, ..., *K*-1, *K* corresponds to the maximum unambiguous range, ${\mathscr {F}}$ is the fast Fourier transform operator, *w(n)* is a rectangular window function in fast time, *w(n)* and *W(k)* are a Fourier pair. Since the rectangular window in frequency domain is a sinc function with gradients close to zero around $2\pi \rho \tau _{i,l}$, the frequency domain window function $W_{i,j}(k)$ can be considered as a fixed one in *slow time*. Assuming *P* subjects and *Q* static clutter in a room, equation (7) can be rewritten as:

8 $$\begin{aligned} X(k,n_{RX},m)= & {} \sum _{i=1}^{P} a_i(n_{RX}) b_i(k) e^{j\phi _i(m)}+a_i(n_{RX})c(k), \end{aligned}$$

9 $$\begin{aligned} a_i(n_{RX})= & {} e^{j\dfrac{2\pi f_0 (n_{RX}-1) \cdot d\sin \alpha _i}{c_0}}, \end{aligned}$$

10 $$\begin{aligned} b_i(k)= & {} \sum _{l} \beta _{i,l}^{*} a_T^{2} W_{i,l}(k)\cdot e^{j\dfrac{4\pi f_0 r_{i,l}}{c_0}}, \end{aligned}$$

11 $$\begin{aligned} \phi _i(m)= & {} \dfrac{4\pi f_0}{c_0}y_i(m), \end{aligned}$$

12 $$\begin{aligned} c(k)= & {} \sum _{i=P+1}^{P+Q} \sum _{l} \beta _{i,l}^{*} a_T^{2} W_{i,l}(k) \cdot e^{j\dfrac{4\pi f_0 r_{i,l}}{c_0}}, \end{aligned}$$

where $\phi _i(m)$ is the Doppler shift caused by the vital signs.

**Figure 2:**
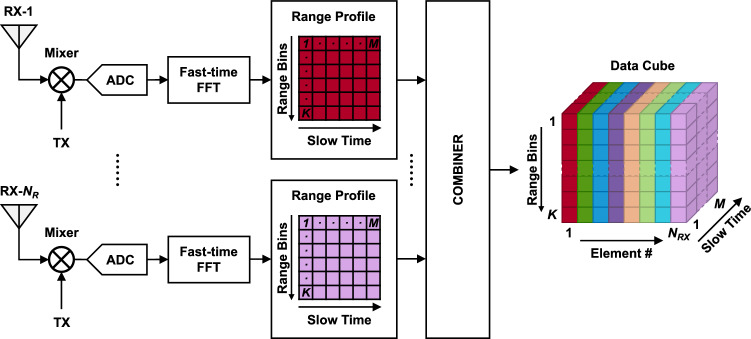
Block diagram of the data cube generation.

The block diagram of the data cube generation is shown in Fig. 2. Per each TX-RX pair, a range profile matrix is created performing a *K*-point FFT to *M* consecutive *beat signals*. The range profile matrices are then stacked together to form the data cube, which is the starting point for the signal processing algorithm described in “Methods”.

## Methods

**Figure 3:**
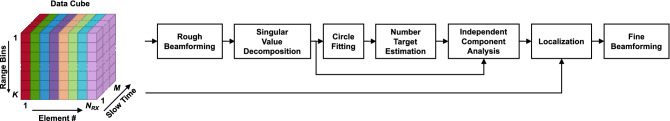
Block diagram of the proposed algorithm. SVD stands for singular value decomposition and ICA stands for independent component analysis.

**Figure 4:**
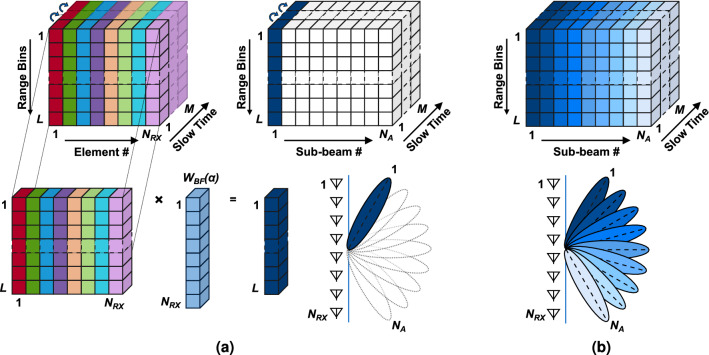
Graphical illustration of the rough beamforming algorithm. (**a**) Vector beamforming using the data cube and a weighting vector of a certain angle. (**b**) Angle data cube obtained after scanning all the angles under study.

The block diagram of the proposed algorithm for automatic detection of the number of human subjects and 2-D localization is shown in Fig. 3. In order to reduce the computational complexity and memory usage, due to the limited size of the room, we consider only the first *L* of the possible *K* range bins.

### Rough beamforming

The conventional beamformer is performed in frequency domain over the data cube described in (8) for all the antenna elements and for each range bin. We obtain the angle data cube which can be expressed as:

13 $$\begin{aligned} X_{angle}(k,n_a,m) = X(:,:,m) \times W_{BF}(\alpha ), \end{aligned}$$

where $n_a$ = 0, ..., $N_A$-1 is the angle index corresponding to a certain angle, $N_A$ is the number of considered angles, and $\times$ indicated the vectorial product operation. The graphical illustration of the beamforming is shown in Fig. 4. From the initial data cube, a sub-matrix, containing all the range bins information of all antennas at a certain *slow time* index, is extracted. It is multiplied by a weighting vector $W(\alpha )$ of $N_{RX}$ element, whose coefficient have been calculated considering a certain angle and using the following formula:

14 $$\begin{aligned} W_{BF}(\alpha ) = \exp \left[ -j\dfrac{2\pi }{\lambda _0}d(n_{RX}-1) \sin \alpha \right] . \end{aligned}$$

This results in an *L*-element vector which is inserted in the angle data cube at the (:,$n_a$,*m*) indexes. This operation is repeated until all the *M* *slow time* indices have been considered. This corresponds to focus the beam to the first angle under study (Fig. 4a). The very same process is then repeated with a next angle, and then with a new weighting vector, until all the angles of the rough beamforming have been covered. This results in the angle data cube depicted in Fig. 4b.

### Static reflectors removal

The sub-matrices of (13) along the $n_a$ dimension can be modelled as:

15 $$\begin{aligned} X_{angle}(:,n_a,:) = \mathbf{X }_A = \mathbf{HS }+\mathbf{C }, \end{aligned}$$

where

16 $$\begin{aligned} \mathbf{H }= \begin{bmatrix} b_1(0) &{} \cdots &{} b_p(0)\\ \vdots &{} \ddots &{} \vdots \\ b_1(L-1) &{} \cdots &{} b_p(L-1) \end{bmatrix} \end{aligned}$$

is an *L* $\times$ *P* complex mixing matrix whose elements are described in equation (10),

17 $$\begin{aligned} \mathbf{S }= \begin{bmatrix} e^{j\phi _1(0)} &{} e^{j\phi _1(1)} &{} \cdots &{} e^{j\phi _1(M-1)}\\ \vdots &{} \vdots &{} \ddots &{} \vdots \\ e^{j\phi _P(0)} &{} e^{j\phi _P(1)} &{} \cdots &{} e^{j\phi _P(M-1)} \end{bmatrix} \end{aligned}$$

is a *P* $\times$ *M* complex matrix containing the Doppler signals equation (11), and hence the vital signs information caused by the *P* subjects at each $T_{s}$, and

18 $$\begin{aligned} \mathbf{C }= \begin{bmatrix} c(0)&\cdots&c(L-1) \end{bmatrix} ^{\mathrm {T}} \cdot \mathbf{1 }^{\mathrm {T}} \end{aligned}$$

is an *L* $\times$ *M* matrix with identical columns containing the direct current (DC) information in *slow time* resulting from static reflections equation (12), the superscript *T* indicates the transpose, and **1** is a length *M* all-ones column vector. In presence of additive noise, the data model becomes as:

19 $$\begin{aligned} \mathbf{X }_A = \mathbf{HS }+\mathbf{C }+\mathbf{N }, \end{aligned}$$

where **N** is an *L* $\times$ *M* matrix containing the additive white Gaussian noise assumed to be independent and identically distributed (I.I.D.) for different range-azimuth bins. In order to remove the DC contribution of the static reflectors (e.g., clutter, static parts of the human body, ...), we perform alternate current (AC) coupling to ’Eq. (19). The latter is achieved subtracting the mean value along the *slow time* for each range and angle. It should be noted that the variable cardiopulmonary signals are preserved. Each sub-matrix can be then expressed as:

20 $$\begin{aligned} \overline{\mathbf{X }}_{angle}(:,n_a,:) = \overline{\mathbf{X }}_A=\mathbf{H }\overline{\mathbf{S }}+\overline{\mathbf{N }}, \end{aligned}$$

where **H** determines the linear combinations of the sources in $\overline{\mathbf{S }}$, so the magnitudes of the elements in **H** indicate the energy of the sources in every range bin.

### Singular value decomposition

The angle data cube $\overline{\mathbf{X }}_{angle}$ is re-arranged in a 2-D matrix $\overline{\mathbf{X }}_{2D}$. This is performed by transposing each of the $N_A$ $\overline{\mathbf{X }}_A$ sub-matrices and then concatenating them by columns. The result is an *M* $\times$ (*L*$\cdot$ $N_A$) modelled as:

21 $$\begin{aligned} \overline{\mathbf{X }}_{2D} = \mathbf{Z }\overline{\mathbf{S }}+\overline{\mathbf{N }}, \end{aligned}$$

where **Z** is the result of the concatenation of the $N_A$ **H** sub-matrices. The observation matrix $\overline{\mathbf{X }}_{2D}$ contains signals which can be direct paths, multipaths, or combinations of them. Those signals are linear combinations of the *P* independent sources **S** which are the information to retrieve.

We use the economy-sized singular value decomposition (SVD) as the first step of the proposed methodology to determine the number of persons *P*. It is applied to Eq. (21) as:

22 $$\begin{aligned} \overline{\mathbf{X }}_{2D}=\mathbf{Z }\overline{\mathbf{S }}+\mathbf{N } =\mathbf{U }\cdot \Sigma \cdot \mathbf{V }^{\mathbf{H }}, \end{aligned}$$

where **U** and **V** are matrices containing singular vectors, $\Sigma$ is a diagonal matrix containing all the singular values, and the superscript *H* indicates the Hermitian transpose. The economy-sized SVD saves both time and storage by producing an *M* $\times$ *M* **U**, an *M* $\times$ *M* $\Sigma$, and an (*L*$\cdot$ $N_A$) $\times$ *M* **V**. The next step is to define a metric to evaluate the most significant components of **U** by which we determine *P*.

### Circle fitting

Before evaluating the most significant components of **U**, we perform phase demodulation in *slow time* (i.e., per columns). Considering the operating wavelengths (i.e., mmWave range) and the typical values of the mechanical displacements of the lungs and the heart, the Doppler signal produced by the cardiopulmonary activity describes a circle in a complex plane^16–18^. For a proper phase demodulation, and hence to avoid distortions, this circle should first be centered to the origin. The AC coupling applied to Eq. (19) (introduced in  “Static reflectors removal”) should already centre the circle to the origin. However, due to the presence of noise, this may not be optimal^44^. Therefore, we propose the following technique which is applied to each column of **U**. The coordinates of the center **z**$_c$ (in the complex plane) and the radius $r_c$ of a circle can be estimated by resolving a nonlinear least-squares geometric fitting problem^45^. Assume ${\mathbf {u}}=\left[ {\mathbf {u}}^R,{\mathbf {u}}^I \right]$ being an *M* $\times$ 2 matrix containing the real (R) and imaginary (I) parts of the complex signal corresponding to a column of **U**, the objective function becomes:

23 $$\begin{aligned} \min \limits _{{\mathbf {z}}_c,r_c} \sum _{m=1}^{M} d_m\left( {\mathbf {z}}_c,r_c\right) ^2, \end{aligned}$$

with

24 $$\begin{aligned} d_m^{2}=\left( \Vert {\mathbf {z}}_c-{\mathbf {u}}(m)\Vert -r_c\right) ^{2}, \end{aligned}$$

representing the geometric distance between the *m*th sample and the circle. The best circle is then iteratively computed. A good starting vector is the solution of minimizing the algebraic distance^45^. The algebraic representation of a circle is defined as:

25 $$\begin{aligned} F\left( {\mathbf {u}} \right) = d{\mathbf {u}}^T{\mathbf {u}}+\mathbf{e }^T{\mathbf {u}}+f=0, \end{aligned}$$

where *d* is a nonzero number and **e** $\in$ $\mathrm{I\!R}^2$. Given ${\mathbf {u}}$, we can compute the circle parameters ${\hat{d}}$, $\hat{\mathbf{e }}$, ${\hat{f}}$. Only when all the samples are on one circle we can find a unique solution. Otherwise, it is an overdetermined problem when the sample length is more than 3. Let $\varvec{\eta }_m = \left[ d,e_1,e_2,f \right] ^T$ and $\mathbf{B }_m = [{\mathbf {u}}_{m}{\mathbf {u}}_{m}^T,{\mathbf {u}}_{m},{\varvec{1}}]$, Eq. (25) can be converted into a linear equation $\mathbf{B }_m \varvec{\eta }_m=\mathbf{0 }$. The non-trival solution can be obtained by solving the following standard optimization problem,

26 $$\begin{aligned} \min \Vert \mathbf{B }_m\varvec{\eta }_m\Vert \, \mathrm {subject\,to\,} \Vert \varvec{\eta }_m\Vert = 1. \end{aligned}$$

The center and the radius can be then computed as:

27 $$\begin{aligned} {\mathbf {z}}_c= & {} \left( z_{1},z_{2} \right) =\left( -\dfrac{e_1}{2d},-\dfrac{e_2}{2d}\right) , \end{aligned}$$

28 $$\begin{aligned} r_c= & {} \sqrt{\dfrac{\Vert \mathbf{e }\Vert ^2}{4d^2}-\dfrac{f}{d}}. \end{aligned}$$

After knowing the coordinates of the center, the circle can be shifted to the origin of the complex plane. As a result, we can demodulate the phase by directly computing the angular information of the complex signal as:

29 $$\begin{aligned} {\hat{\phi }}(m) = \arctan \left( \dfrac{{\mathbf {u}}^I(m)-z_{2}}{{\mathbf {u}}^R(m)-z_{1}}\right) . \end{aligned}$$

This operation is applied to all the *M* columns of ${\mathbf {U}}$, obtaining *M* ${\hat{\phi }}(m)$. This method works well also with radio-frequency (RF) radars, where the vital signs information describes an arc in the complex plane.

### Target number estimation

The spectrum of a canonical vital signs signal extracted using radar techniques consists mainly of the respiration fundamental, some respiration harmonics, decreasing in magnitude (normally up to three), and the very small heartbeat fundamental. The signals also contain the heartbeat harmonics, however their magnitudes are so weak that they can be neglected. The energy of the signal is essentially contained in the fundamental and first harmonic of the respiration. We determine *P* by calculating the signal-to-noise (SNR) of the *M* ${\hat{\phi }}(m)$. We estimate the signal power considering the spectrum within the respiration fundamental and its first harmonic, while the remaining spectrum is used to determine the noise power. We also perform an additional check on the spectra’s local maxima. First, if the main peak, which in a canonical spectrum indicates the respiration rate, is outside the typical medical ranges of 0.1-0.4 Hz, we conclude that this source is noise. Secondly, we determine the magnitude ratio of the strongest peak (expected to be the respiration fundamental) and its first harmonic. We assume as noise any source with a ratio less than 2. The latter comes with the observation that the respiratory physiology involves signals consisting of a dominant fundamental and smaller (more than half) harmonics^19, 33, 34^. In the two aforementioned situations, we set the SNR to the minimum of -20 dB. The SNR values are stored in a vector, whose *n*-th element is related to the *n*-th uncorrelated sources in **U**. The final step is to scan this vector starting from the first position. We stop before the first value is below a threshold which, in this work, was determined empirically and set to 10 dB. We assign the corresponding index to *P*. We denote the first *P* sources of **U** as $\mathbf{U} _{S}$.

### Independent component analysis

The independent component analysis (ICA) allows separating the statistically independent sources $\overline{\mathbf{S }}$ from the set of observations $\mathbf{U} _S$. The latter are a linear combination of the sources and they can be expressed as:

30 $$\begin{aligned} {\mathbf {U}}_S=\mathbf{A }\overline{\mathbf{S }}, \end{aligned}$$

where **A** is called mixing matrix. Therefore, knowing the $\mathbf{U} _S$, it is possible to determine $\hat{\mathbf{S }}$ by evaluating the unmixing matrix, namely $\hat{\mathbf{A }}^{-1}$, as:

31 $$\begin{aligned} \hat{\mathbf{S }}=\hat{{\mathbf {A}}}^{-1}{\mathbf {U}}_S. \end{aligned}$$

Hence, the ICA determines $\hat{\mathbf{S }}$ estimating $\hat{\mathbf{A }}^{-1}$, while $\mathbf{U} _S$ is provided by the SVD. The sources $\hat{\mathbf{S }}$ are the vital signs signatures we use to localize the subjects.

### 2-D localization

The 2-D localization is performed on the angle data cube $\overline{\mathbf{X }}_{angle}$. More precisely, to each of its sub-matrices $\overline{\mathbf{X }}_{A}$, we estimate $\hat{\mathbf{H }}$, which contains the energy of the sources in every range bin (i.e., the channel information), by minimizing the residual error, as:

32 $$\begin{aligned} \min \limits _{\overline{\mathbf{H }}} \Vert \overline{\mathbf{X }}_A-\mathbf{H }\hat{\mathbf{S }} \Vert _{2}^{2} + \zeta \Vert \mathbf{H }\Vert _{1}, \end{aligned}$$

where $\zeta$ is the penalty coefficient which represents a trade-off between the residual error and the sparsity, whose value was determined empirically. The results of this operation are $N_{A}$ $\hat{\mathbf{H }}$ propagation channels, one per each $n_a$-th considered angle. The final results are hence the responses of each subject (i.e., source) in every azimuth-range bin from which it is possible to perform the 2-D localization (example in “Results and discussion”).

Once the subjects have been 2-D located, it is possible to improve the accuracy of the azimuth information through a fine beamforming. This means re-running the proposed algorithm and performing the conventional beamformer only around the angles where the subjects were detected during the rough beamforming with a fine angular step. The beamforming algorithms are generally computationally heavier, that’s why the fine beamforming is not applied directly as first option.

## Results and discussion

We conducted the experimental validation using the commercial Texas Instruments IWR6843ISK mmWave radar sensor, configured to operate as a SIMO FMCW architecture with a ULA antenna array of 4 elements. The distance between the TX antenna and the adjacent RX antenna is of 5 mm. Therefore, we can safely assume co-located radar. The system parameters are: ${f_0} = 60.645$ GHz, $\textit{B} = 3.25$ GHz, $\textit{T} = 64$ $\mu$s, PRI = 50 ms and ${T_{f}} = 0.25$ $\mu$s. We implemented the algorithm in MATLAB. In order to reduce the spectral leakage, before applying the FFT in *fast time*, each *beat signal* is multiplied by a Hann window function. We fixed *L* equal to 111, corresponding to a maximum range of 4.5 m (the range resolution is of 4.05 cm). All procedures in this study protocol adhered to the ethical principles of the Declaration of Helsinki. Written informed consent was provided by all patients before they were enrolled in the study. The IMEC ethical board reviewed and approved the study protocols (IP-19-WATS-TIP2-056). All the collected data were pseudonymized.

**Figure 5:**
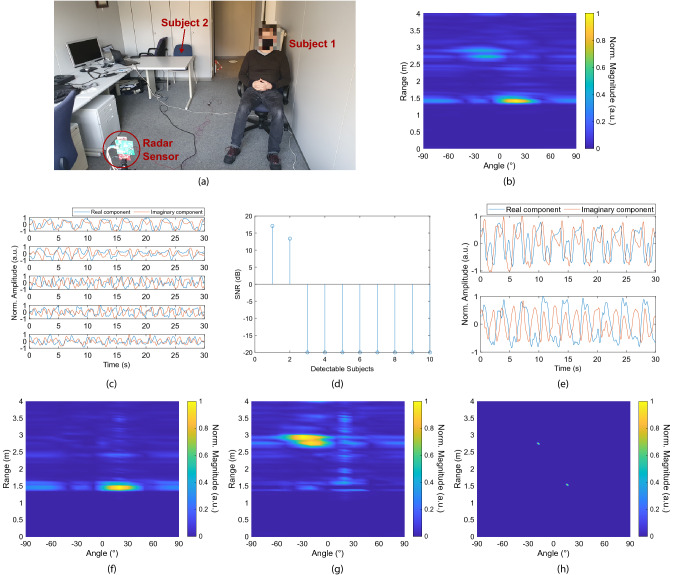
Experiment with two seated and normally breathing subjects at 1.5 m / 16.85$^{\circ }$ and 2.7 m / -18.71$^{\circ }$ away from the radar. (**a**) Experimental environment. Subject 2 took the picture. (**b**) 2-D map after the rough beamforming. (**c**) First five components of the SVD. (**d**) Result of the target number estimation operation. (**e**) Estimated independent sources (i.e., vital signs signature). (**f**) Responses of Subject 1. (**g**) Responses of Subject 2. (**h**) 2-D localization after the fine beamforming. An angular step of 10$^{\circ }$ was used for (**b**), (**f**), (**g**) and one of 2$^{\circ }$ for (**h**). Interpolation was performed to obtain the 2-D maps.

**Table 1:** Results of the experimental validation.

Test	Expected results				Measured results			
	Subject 1	Subject 1	Subject 2	Subject 2	Subject 1	Subject 1	Subject 2	Subject 2
	Distance (m)	Angle ($^{\circ }$)	Distance (m)	Angle ($^{\circ }$)	Distance (m)	Angle ($^{\circ }$)	Distance (m)	Angle ($^{\circ }$)
1	1.5	$-$18.43	1.5	18.43	1.42	$-$16	1.42	24
2	1.5	$-$18.43	1.5	18.43	1.42	$-$18	1.46	18
3	1.5	$-$18.43	2.5	11.31	1.46	$-$14	2.35	10
4	1.5	$-$18.43	2.5	11.31	1.42	$-$18	2.39	10
5	2.5	$-$11.31	2.5	11.31	2.47	$-$12	2.31	10
6	2.5	$-$11.31	2.5	11.31	2.31	$-$18	2.31	10
7	2.5	$-$11.31	1.5	18.43	2.31	$-$14	1.46	16
8	2.5	$-$11.31	1.5	18.43	2.31	$-$12	1.42	16
9	2.5	$-$11.31	4	6.34	2.31	$-$12	4.05	10
10	2.5	$-$11.31	4	6.34	2.31	$-$12	4.05	10
11	4	$-$6.34	2.5	11.31	3.89	$-$10	2.35	12
12	4	$-$6.34	2.5	11.31	3.97	$-$10	2.35	12
13	2.7	$-$18.71	1.5	16.85	2.67	$-$18	1.5	16
14	2.7	$-$18.71	1.5	16.85	2.75	$-$18	1.54	16
15	3	$-$17.94	1.8	20	3.04	$-$16	1.78	20
16	3	$-$17.94	1.8	20	3.04	$-$24	1.86	20

**Table 2:** Mean absolute errors reported in this validation.

	T1 distance	T1 angle		T2 distance	T2 angle	
	(m)	($^{\circ }$)	(m)	(m)	($^{\circ }$)	(m)
MAE	0.1	2.29	0.11	0.09	1.7	0.07
RMSE	0.12	3.04	0.15	0.1	2.24	0.11

**Table 3:** Performance comparison of this work with alternative state-of-the-art radars.

Ref. no.	Radar type	Needs info no. of subj.	Multipath rejection capab.	Max distance (m)	Range capab.	Max range error (m)	Ang. meas. capab.	Max ang. error ($^{\circ }$)	Min. ang. separation ($^{\circ }$)
^33^$$^{*}$$	CW SIL	Yes	No	3.3	Yes	0.04	Yes	5	15
^34^$$^{*}$$	SIMO CW	Yes	No	3	No	N.A.	Yes	3	30
^35^	MIMO CW	Yes	No	1.8	No	N.A.	Yes	2	17
^37^	MIMO FMCW	No	No	3	Yes	0.4	Yes	8	30
^46^	Scan. FMCW	Yes	No	4.3	Yes	0.066	Yes	N.A.	N.A
^47^	SIMO FMCW	Yes	No	3	Yes	N.A.	Yes	N.A.	30
This work	SIMO FMCW	No	Yes	4	Yes	0.19	Yes	6	18

$^{*}$Based on a single experiment.

Figure 5 shows the results of an experiment with two volunteers in an office room whose 2-D positions are 1.5 m / 16.85$^{\circ }$ and 2.7 m / -18.71$^{\circ }$, respectively (Fig. 5a). We used a measuring tape to determine the absolute distances between the radar and middle of the chest area of the subjects (expected results). We determined the azimuth information using geometric calculation. The radar position was considered as the origin, namely 0 m/0$^{\circ }$ in the polar coordinate system. The orientation was calculated considering the line of sight (LoS) of the radar as 0$^{\circ }$. Clockwise angles were treated as positive while counterclockwise ones as negative. Subject 1 (the closest to radar) is seated right next to a metal wall, while Subject 2 (the farthest to radar) is seated in between an office desk and a large metal cabinet. This clutter causes a strong spreading of the transmitted and reflected signals in the whole room, generating significant multipaths. Due to the closer proximity to the radar, the Subject 1 is not affected by the multipaths of Subject 2 but their Doppler signal is strongly influenced by the metal wall (very strong reflector). Furthermore, the latter might reflect the multipath signals generated by Subject 1 which can have identical delays (time of flight) as the direct path signal of Subject 2, involving non-linear combinations of their phase contents. This generates uncorrelated signals that can be interpreted as independent targets (i.e., radar ghosts). Figure 5b shows the 2-D map (range vs. angle) obtained after the rough beamforming (we considered angular steps of 10$^{\circ }$). It is possible to see the strong contribution of Subject 1, two significant responses nearby the expected location of Subject 2, and other effects due to the multipath, side lobes, and FFT spreading. In such a situation, it is not trivial to determine the right number of subjects in the room and their positions. It should be noted that, depending on the radar cross section (RCS), the multipath signal generated by a closer target might appear much stronger than the direct path of a distant target. Figure 5c shows the first five components produced by the SVD. As expected, the latter cannot provide exclusively the two vital signs signatures. In fact, we can clearly see periodicities in these five components which may resemble vital signs signals. Figure 5d shows the output of the target number estimation operation, from which only the first two components of the SVD are considered valid. This sets *P* $=$ 2. These signals are then processed by the ICA, whose results are shown in Fig. 5e. At this point, we face an ordering ambiguity issue. We are still not able to indicate which source (i.e., vital signs signature) corresponds to which subject. We solve this with the 2-D localization operation, where we determine the responses of each source (i.e., target), as shown in Fig. 5f,g. In Fig. 5g, it is possible to see the subject’s response and the very strong multipath effect. We remove the outliers resulting from multipaths by detecting the shortest direct path, while the outliers originating from additive noise are small and can easily be excluded. We finally perform the fine beamforming with angular steps of 2$^{\circ }$. The final 2-D localization map is shown in Fig. 5h. Subject 1 was localized at 1.54 m/16$^{\circ }$ while Subject 2 at 2.75 m/−18$^{\circ }$. These results are in fair agreement with the expected values.

We validated the proposed approach conducting experiments on 6 subjects, differing in height (170–195 cm), in weight, and in age (30–50 years). The subjects were seated on chairs with their chest regions facing the radar. Furniture and objects were present near and in between the volunteers, who were grouped in random pairs and they could randomly chose a seat. Two measurements have been collected at the same 2-D location. The reference values for the distances and for the angles where the subjects were expected to be located are reported in Table 1 under “Expected Results”. For the first 12 experiments, we considered 3 absolute distances (ranges) with the subjects’ chest centers at 0.5 m away from the LoS of the radar. For the remaining experiments, we selected random positions in the room. The experimental results, reported in Table 1 under “Measured Results”, demonstrate that the proposed approach was able to accurately determine the 2-D location of the subjects. In Table 2, we reported as maximum mean absolute errors (MAEs) 0.1 m and 2.29$^{\circ }$ and as maximum root-mean-square errors (RMSEs) 0.12 m and 3.04$^{\circ }$ in measuring respectively the ranges and azimuth angles. Converted to meters, the maximum angular errors corresponds to 0.11 m for the MAE and 0.15 for the RMSE. Compared to the typical size of the human bodies, these small errors can be considered acceptable. Finally, in Table 3, we compared some relevant state-of-the-art works for automatic localization.

### Conclusion

In this work, we proposed a signal processing algorithm, demonstrated using a mmWave SIMO FMCW radar, to automatically determine the number and the 2-D positions of human subjects. This method aims at separating the radar reflections (direct paths from multipaths) to retrieve the vital signs signatures of the subjects present in the monitored environment. We determine the number of people by counting the number of sensed cardiopulmonary activities, while the 2-D localization is performed by measuring the distances from the radar to the thoracic regions of the subjects. The experimental validation has proven the ability of the proposed approach in monitoring people in a typical office room, reporting maximum MAES of 0.1 m and 2.29$^{\circ }$ and maximum RMSEs of 0.12 m and 3.04$^{\circ }$ in measuring respectively the ranges and azimuth angles. This radar system and signal processing technique can be considered a useful technology for the development of future telemedicine.

## Data Availability

The data that support the plots within this paper and other findings of this study are available from the corresponding author upon reasonable request.
